# Diagnostic Investigation of 100 Cases of Abortion in Sheep in Uruguay: 2015–2021

**DOI:** 10.3389/fvets.2022.904786

**Published:** 2022-05-19

**Authors:** Matías A. Dorsch, María E. Francia, Leandro R. Tana, Fabiana C. González, Andrés Cabrera, Lucía Calleros, Margarita Sanguinetti, Maila Barcellos, Leticia Zarantonelli, Camila Ciuffo, Leticia Maya, Matías Castells, Santiago Mirazo, Caroline da Silva Silveira, Ana Rabaza, Rubén D. Caffarena, Benjamín Doncel Díaz, Virginia Aráoz, Carolina Matto, Joaquín I. Armendano, Sofía Salada, Martín Fraga, Sergio Fierro, Federico Giannitti

**Affiliations:** ^1^Plataforma de Investigación en Salud Animal, Instituto Nacional de Investigación Agropecuaria (INIA), Estación Experimental La Estanzuela, Colonia, Uruguay; ^2^Laboratorio de Biología de Apicomplejos, Instituto Pasteur de Montevideo, Montevideo, Uruguay; ^3^Departamento de Parasitología y Micología, Facultad de Medicina, Universidad de la República, Montevideo, Uruguay; ^4^Laboratorio de Interacciones Hospedero-Patógeno, Instituto Pasteur de Montevideo, Montevideo, Uruguay; ^5^Unidad de Microbiología, Departamento de Patobiología, Facultad de Veterinaria, Universidad de la República, Montevideo, Uruguay; ^6^Sección de Genética Evolutiva, Facultad de Ciencias, Universidad de la República, Montevideo, Uruguay; ^7^Unidad Mixta Instituto Pasteur de Montevideo e Instituto Nacional de Investigación Agropecuaria (UMPI), Montevideo, Uruguay; ^8^Laboratorio de Virología Molecular, Departamento de Ciencias Biológicas, Centro Universitario Regional (CENUR) Litoral Norte, Universidad de la República, Salto, Uruguay; ^9^Laboratorio de Virología, Facultad de Ciencias, Universidad de la República, Montevideo, Uruguay; ^10^Departamento de Bacteriología y Virología, Instituto de Higiene, Facultad de Medicina, Universidad de la República, Montevideo, Uruguay; ^11^Unidad Académica Salud de los Rumiantes, Departamento de Producción Animal, Facultad de Veterinaria, Universidad de la República, Montevideo, Uruguay; ^12^Laboratorio de Patología Veterinaria, Facultad de Medicina Veterinaria y de Zootecnia, Universidad Nacional de Colombia, Sede Bogotá, Bogotá, Colombia; ^13^Laboratorio Regional Noroeste, División de Laboratorios Veterinarios (DILAVE) Miguel C. Rubino, Ministerio de Ganadería, Agricultura y Pesca (MGAP), Paysandú, Uruguay; ^14^Facultad de Ciencias Veterinarias, Universidad Nacional del Centro de la Provincia de Buenos Aires (UNCPBA), Tandil, Argentina; ^15^Secretariado Uruguayo de la Lana (SUL), Montevideo, Uruguay

**Keywords:** sheep, abortion, pathology, toxoplasmosis, campylobacteriosis, dystocia, reproductive losses, infectious diseases

## Abstract

The aim of this work was to identify causes of abortion through laboratory investigations in sheep flocks in Uruguay. One hundred cases of abortion, comprising 58 fetuses, 36 fetuses with their placentas, and 6 placentas were investigated in 2015–2021. Cases were subjected to gross and microscopic pathologic examinations, and microbiological and serological testing for the identification of causes of abortion, including protozoal, bacterial, and viral pathogens. An etiologic diagnosis was determined in 46 (46%) cases, including 33 (33%) cases caused by infectious pathogens, as determined by the detection of a pathogen along with the identification of fetoplacental lesions attributable to the detected pathogen. Twenty-seven cases (27%) were caused by *Toxoplasma gondii*, 5 (5%) by *Campylobacter fetus* subspecies *fetus*, and 1 (1%) by an unidentified species of *Campylobacter*. Fourteen cases (14%) had inflammatory and/or necrotizing fetoplacental lesions compatible with an infectious etiology. Although the cause for these lesions was not clearly identified, *T. gondii* was detected in 4 of these cases, opportunistic bacteria (*Bacillus licheniformis, Streptococcus* sp.) were isolated in 2 cases, and bovine viral diarrhea virus 1 subtype i (BVDV-1i) was detected in another. *Campylobacter jejuni* was identified in 1 (1%) severely autolyzed, mummified fetus. BVDV-2b was identified incidentally in one fetus with an etiologic diagnosis of toxoplasmosis. Microscopic agglutination test revealed antibodies against ≥1 *Leptospira* serovars in 15/63 (23.8%) fetuses; however, *Leptospira* was not identified by a combination of qPCR, culture, fluorescent antibody testing nor immunohistochemistry. *Neospora caninum, Chlamydia abortus, Chlamydia pecorum, Coxiella burnetii* and border disease virus were not detected in any of the analyzed cases. Death was attributed to dystocia in 13 (13%) fetuses delivered by 8 sheep, mostly from one highly prolific flock. Congenital malformations including inferior prognathism, a focal hepatic cyst, and enterohepatic agenesis were identified in one fetus each, the latter being the only one considered incompatible with postnatal life. Toxoplasmosis, campylobacteriosis and dystocia were the main identified causes of fetal losses. Despite the relatively low overall success rate in establishing an etiologic diagnosis, a systematic laboratory workup in cases of abortion is of value to identify their causes and enables zoonotic pathogens surveillance.

## Introduction

The economy of Uruguay is mainly based on agriculture and livestock production (1), and the ovine sector is responsible for a significant proportion of the gross domestic product generated by the livestock sector through the production, industrialization, internal commercialization and export of wool, skins, meat and cheese (2). Despite its economic relevance, sheep stocks have dropped significantly over the past decades, mainly because of political, economic, sanitary and environmental factors (3). This downward trend is further aggravated by the overall low (re)productive efficiency of Uruguayan flocks (4, 5). In this context, reproductive diseases account for significant economic losses; they negatively affect income by reducing the number of weaned lambs and rise production costs by increasing the culling rate and replacement of aborted ewes, increasing the costs of feeding non-productive ewes, labor, and veterinary services, among other costs (4).

Among the etiologies of ovine abortion, defined as fetal death followed by expulsion, there are infectious agents, including several zoonotic pathogens, and non-infectious causes (i.e., nutritional, genetic, metabolic, toxic, and physical -dystocia, trauma, injury-) (6, 7). Although the occurrence and prevalence of abortigenic pathogens vary with the geographic region, it is widely accepted that zoonotic pathogens such as *Toxoplasma gondii, Campylobacter* spp., *Chlamydia abortus*, and *Coxiella burnetii* are amongst the most frequent infectious causes of abortion in sheep (8–10). Furthermore, many abortigenic pathogens of sheep are notifiable to the World Organization of Animal Health (OIE) as their occurrence may imply restrictions to the international trade of livestock or animal products (11). As such, identifying ovine abortigenic pathogens is key to developing strategies to control and prevent reproductive losses, monitor endemic, exotic, (re)emerging and transboundary diseases, and reduce public health risks (12).

Monitoring programs for the detection of ovine abortigenic pathogens have been conducted in several European and Oceanic countries with a long-standing tradition in sheep production (8, 12–20). Such programs have not been implemented systematically in Uruguay or other South American countries, thus the current understanding on the etiology of abortion in local and regional flocks is limited (21). In Uruguay, only *T. gondii* and more recently individual cases of *Campylobacter* spp. have been confirmed as causes of ovine abortion (22–24). Additionally, there is serological evidence of exposure to *Leptospira* spp. (25), *Neospora caninum* (26) and *C. abortus* (27). Although the amount of evidence is limited, it suggests that several diseases may negatively affect the reproductive performance of local flocks. Given this, there is a gap of knowledge concerning the causative agents of abortion. Identifying the involved abortifacients is the first step that must be undertaken to implement future control and prevention programs. With this in mind, herein we aim to identify the causes of abortion in sheep flocks in Uruguay at the laboratory level through pathological, bacteriological, molecular and serological assays.

## Materials and Methods

### Case Selection

Between July 2015 and August 2021, 100 cases of ovine abortion were processed at the veterinary diagnostic laboratory of the “Plataforma de Investigación en Salud Animal” of INIA La Estanzuela. Cases were submitted from 30 private commercial and 4 experimental farms. The latter are owned by the “Secretariado Uruguayo de la Lana” (SUL) (2 flocks), “Facultad de Agronomía de la Universidad de la República” (UdelaR) (1 flock), or INIA La Estanzuela (1 flock).

Cases included 3 different types of submissions: 1—aborted fetuses without their placenta, 2—aborted fetuses with their placenta, or 3—placentas of aborted sheep without fetuses. More than one fetus and/or placentas without fetuses expulsed by the same sheep were considered different cases. For instance, on one occasion one ewe expulsed 3 fetuses and 1 placenta with no clear association with any of the fetuses; thus, all 4 specimens were considered individual cases.

### Pathologic Examination and Sample Collection

All fetuses were autopsied, and all placentas were examined macroscopically to assess for gross lesions. The gestational age was estimated in the autopsied fetuses using the crown-to-rump length, and other fetal developmental features, such as the presence/absence of a developed wool/hair coat, testicular descent into the scrotum in males, and the eruption of the incisor teeth (28). Additional features, such as the degree of autolysis/postmortem decomposition (minimal, mild, moderate, or severe), sex of the fetus, and abnormalities (such fetal mummification or congenital malformations) were recorded. In all cases, tissue and fluid samples (see below) were collected for histological, bacteriological, molecular, and/or serological testing.

### Histology and Immunohistochemistry

Tissue samples collected for histologic examination included brain, liver, lungs, kidneys, heart, spleen, thymus, skeletal muscle, tongue, esophagus, trachea, adrenal glands, abomasum, forestomachs, small and large intestines, and placenta. Samples were fixed in 10% neutral buffered formalin for 48–72 h, routinely processed, embedded in paraffin, cut into 4–5 μm sections, mounted on glass slides, and stained with hematoxylin and eosin. Slides were examined under an optic microscope (AxioScopeA.1, Carl Zeiss, Germany) equipped with a digital color camera (Axiocam 512, Carl Zeiss, Germany) commanded by the ZEN software (Carl Zeiss, Germany).

Selected formalin-fixed paraffin-embedded tissue sections were additionally processed by immunohistochemistry (IHC) for the detection of bovine viral diarrhea virus (BVDV) and *Leptospira* spp., as deemed appropriate on a case-by-case basis, following previously described procedures (29, 30). Additional details of the IHC procedures are provided in Supplementary Materials.

### Bacteriological Cultures

Fresh samples of liver, lung, abomasal fluid, and placenta (when available) were inoculated in blood and MacConkey agars (Oxoid, Basingstoke, Hampshire, England) and incubated in an aerobic atmosphere at 37°C following previous methodologies (31). The same samples were inoculated on Skirrow agar (Oxoid, Basingstoke, Hampshire, England) and subsequently incubated under microaerobic conditions generated using commercial sachets (CampyGen, CN0025, Oxoid, Basingstoke, Hampshire, England) at 37°C. Bacterial isolates were identified by colony morphology, Gram stain, and routine biochemical tests. For leptospiral culture, samples of abomasal fluid, liver, kidney, and placenta were inoculated in Ellinghausen-McCullough-Johnson-Harris (EMJH) medium supplemented with 5-fluorouracil and incubated at 29°C (32). The inoculated media were examined weekly by darkfield microscopy for 6 months.

### Direct Fluorescent Antibody Tests for *Campylobacter fetus* and *Leptospira* spp.

Imprints of fresh placenta, liver, lung, and smears of abomasal fluid were assessed by direct fluorescent antibody testing (DFAT) (31). Slides were fixed in ethanol at room temperature and then incubated with a specific *C. fetus* antiserum (Biotandil, Tandil, Buenos Aires, Argentina) conjugated with fluorescein isothiocyanate (FITC). In addition, DFAT was performed on kidney, liver, abomasal fluid, and placenta imprints/impression smears using a specific multivalent rabbit antiserum bound to FITC (LEP-FAC, NVSL, Ames, Iowa, USA) for *Leptospira* spp. detection. The slides were visualized under a fluorescence microscope (AxioLab.A1, Carl-Zeiss, Germany) set at wavelengths of 495 nm excitation and 519 nm emission.

### Molecular Testing for the Detection of Bacterial, Protozoal, and Viral Pathogens

Nucleic acid (DNA and/or RNA) extraction was performed from frozen fetal tissues, abomasal fluid and/or placentas using different commercial kits, following the manufacturer's instructions. Nucleic acids extracted from different samples (Table 1) were processed by real-time PCR for the detection of *Campylobacter* spp. including species-specific protocols for *C. fetus* and *C. jejuni* (33, 34), a conventional PCR for the simultaneous detection of *C. abortus, Chlamydia pecorum*, and *C. burnetii* (35), a SYBR Green based real-time qPCR for the detection and quantification of *Leptospira* spp. targeting the *lipL32* gene (36), three conventional PCRs for the individual detection of *N. caninum* (37), *T. gondii*, and the nuclear small subunit of the ribosomal RNA (nss-rRNA) of Apicomplexa Coccidia (38), and a reverse transcriptase conventional PCR for the detection of Pestivirus (39, 40). The samples, nucleic acid extraction kits, primers, and reaction conditions for each PCR protocol, as well as the performing laboratory are summarized in Table 1.

**Table 1:** Samples, nucleic acid extraction kits, primers, and reaction conditions used for the PCR protocols for the detection of bacterial, protozoal, and viral pathogens in cases of ovine abortion.

**Agent**	**Samples (extraction kits)**	**Primers**	**Annealing temperature (°C)**	**Cycles**	**Amplicon size (bp)**	**References**
*Campylobacterfetus* ^a^	Liver, kidney, abomasal fluid, placenta (PL)	F: 5′-GCACCTGTCTCAACTTTC-3′ R: 5′-CCTTACCTGGGCTTGAT-3′	60	40	78	(33)
*Campylobacterjejuni* ^a^	Liver, kidney, abomasal fluid, placenta (PL)	F: 5′-TGCACCAGTGACTATGAATAACGA-3′ R: 5′-TCCAAAATCCTCACTTGCCATT-3′	60	40	124	(34)
*Chlamydiaabortus* ^b^	Placenta, lung, liver (MM)	F: 5′-CTCACCATTGTCTCAGGTGGA-3′ R: 5′-ACCGTAATGGGTAGGAGGGGT-3′	61	35	821	(35)
*Chlamydiapecorum* ^b^	Placenta, lung, liver (MM)	F: 5′-TTCGACTTCGCTTCTTACGC-3′ R: 5′-TGAAGACCGAGCAAACCACC-3′	61	35	526	(35)
*Coxiellaburnetii* ^b^	Placenta, lung (MM)	F: 5′-TATGTATCCACCGTAGCCAGT-3′ R: 5′-CCCAACAACACCTCCTTATTC-3′	61	35	687	(35)
*Leptospira* spp.^c^	Placenta, kidney, liver, abomasal fluid (PL)	F: 5′-TAAAGCCAGGACAAGCGCC-3′ R: 5′-TACGAACTCCCATTTCAGCG-3′	60	40	102	(36)
*Neosporacaninum* ^d^	Placenta, brain (QD)	F: 5′-CAGTCAACCTACGTCTTC-3′ R: 5′-GTGCGTCCAATCCTGTAA-3′	55	35	306	(37)
*Toxoplasmagondii* ^d^	Placenta, brain (QD)	F: 5′-CGCTGCAGACACAGTGCATCTGGATT-3′ R: 5′-CCCAGCTGCGTCTGTCGGGAT-3′	60	35	500	(38)
Apicomplexa Coccidia^d^	Placenta, brain (QD)	F: 5′-AAGTATAAGCTTTTATACGGCT-3′ R: 5′-CACTGCCACGGTAGTCCAATAC-3′	56	35	300	(38)
Pestivirus^e^	Pool of liver, kidney, lung, thymus, heart, and spleen (MM)	F: 5′-ATGCCCWTAGTAGGACTAGCA-3′ R: 5′-WCAACTCCATGTGCCATGTAC-3′	60	40	288	(39, 40)

*Bp, base pairs; PL, PureLink^TM^ Genomic DNA Mini Kit (Thermo Fisher Scientific, USA); MM, MagMAX^TM^ Pathogen RNA/DNA Kit (Thermo Fisher Scientific, USA); QD, Quick DNA^TM^ Tissue/Insect Miniprep Kit (Zymo Research, USA); F, forward; R, reverse*.

a *Tests performed at the “Sección de Genética Evolutiva, Facultad de Ciencias, Universidad de la República, Uruguay*.

b *Tests performed at the “Plataforma de Investigación en Salud Animal, Instituto Nacional de Investigación Agropecuaria (INIA), La Estanzuela, Colonia, Uruguay”*.

c *Test performed at the “Unidad Mixta Institut Pasteur de Montevideo-INIA, Uruguay”*.

d *Tests performed at the “Laboratorio de Biología de Apicomplejos, Institut Pasteur de Montevideo, Uruguay”*.

e *Test performed at the “Laboratorio de Virología Molecular, Centro Universitario Regional Norte, Universidad de la República, Uruguay”*.

Additionally, when a positive result was obtained by the mentioned qPCR protocol for *Leptospira* spp. in any sample of a given case, DNA was extracted from all the available samples of the same case using a commercial kit (MagMAX^TM^ Pathogen RNA/DNA Kit, Thermo Fisher Scientific, USA) and processed by a second qPCR protocol targeting the same gene (*lipL32*) but using a TaqMan^TM^ probe (41) instead of SYBR Green. This procedure was performed at INIA La Estanzuela. In the case of Pestivirus, when a positive PCR result was obtained, the 5′UTR genomic region was sequenced (Macrogen, Seoul, South Korea) and phylogenetic analyses were performed to identify the viral species and subtype (39).

### Serologic Testing

The microscopic agglutination test (MAT) for detection and titration of total antibodies against *Leptospira* spp. was performed on fluid (serum) obtained from the fetal thoracic or pericardial cavities (when available), following a previously described procedure (42). Antigens used for MAT included a collection of reference *Leptospira* strains of the serogroups Canicola, Ballum, Grippotyphosa, Icterohaemorrhagiae, Pomona, Tarassovi and Sejroe, as well as a series of autochthonous isolates representative of serovars circulating in Uruguay, including Sejroe, Pyrogenes, Australis, Autumnalis and Pomona. The cut-off point was 1:10, positive samples were further diluted and tested for end-point titration (35, 36). This assay was performed at the “Unidad Mixta Instituto Pasteur de Montevideo-INIA” (UMPI).

### Interpretation of Test Results and Etiologic Diagnosis

The results of the gross and microscopic pathologic examinations and bacteriological, molecular, and serologic testing were integrated and interpreted on a case-by-case basis. An etiologic diagnosis in cases with an infectious cause was reached based on the association between the pathogen identified by bacteriological and/or molecular testing and compatible gross and/or microscopic lesions attributable to the identified pathogen. Cases with an identified pathogen but with no compatible lesions, and cases with lesions and no identified pathogen were considered as cases of undetermined etiology. Serologic testing (MAT) was used as an indirect way to assess for in utero exposure to *Leptospira* spp.; a positive result to this test alone was not considered evidence of abortion causality.

The criteria applied to establish an etiologic diagnosis of fetal death attributed to dystocia included: fetal age near the term of gestation with fully developed fetuses, lack of fetal mummification, traumatic gross lesions such as fractured ribs, hemothorax, hemopericardium or hemoperitoneum along with generalized tissue pallor consistent with anemia due to exsanguination, subcutaneous edema involving the head/neck, wool/hair coat extensively stained with meconium suggesting fetal stress, and lack of relevant histologic lesions in the fetal tissues/placenta. If a diagnosis of dystocia was made in a fetus expulsed by a sheep carrying multiple fetuses submitted to the laboratory, dystocia was also diagnosed in the siblings if they were full-term fetuses with no mummification, no lesions and no pathogens identified.

### Statistical Analyses

Tests for comparison of proportions (Chi square test of independence or Fisher's exact test depending on the number of cases), were used to assess differences (*p* < 0.05) in the cases with an etiologic diagnosis by type of submission (fetuses, fetuses with placentas, placentas without fetuses), degree of autolysis (minimal/mild, moderate, severe), and by state of mummification in fetuses. Additionally, to assess the role of the degree of autolysis (as an ordinal variable), the availability of placenta, and fetal mummification as predictors of the etiologic diagnosis category (infectious etiology, dystocia, undetermined etiology), a conditional inference tree (CTREE) was constructed in R v4.1.2 (R Core Team, 2021) using the packages “party” v1.3-9 and “caret” v6.0-90 (43–45). To fit the CTREE a minimum criterion of 0.55 was selected based on a 10-times repeated 10-fold cross-validation procedure. Missing values in 11 of the records were handled by using surrogate variables. The Bonferroni correction was used to adjust for multiplicity.

## Results

### Case Description

**Figure 1:**
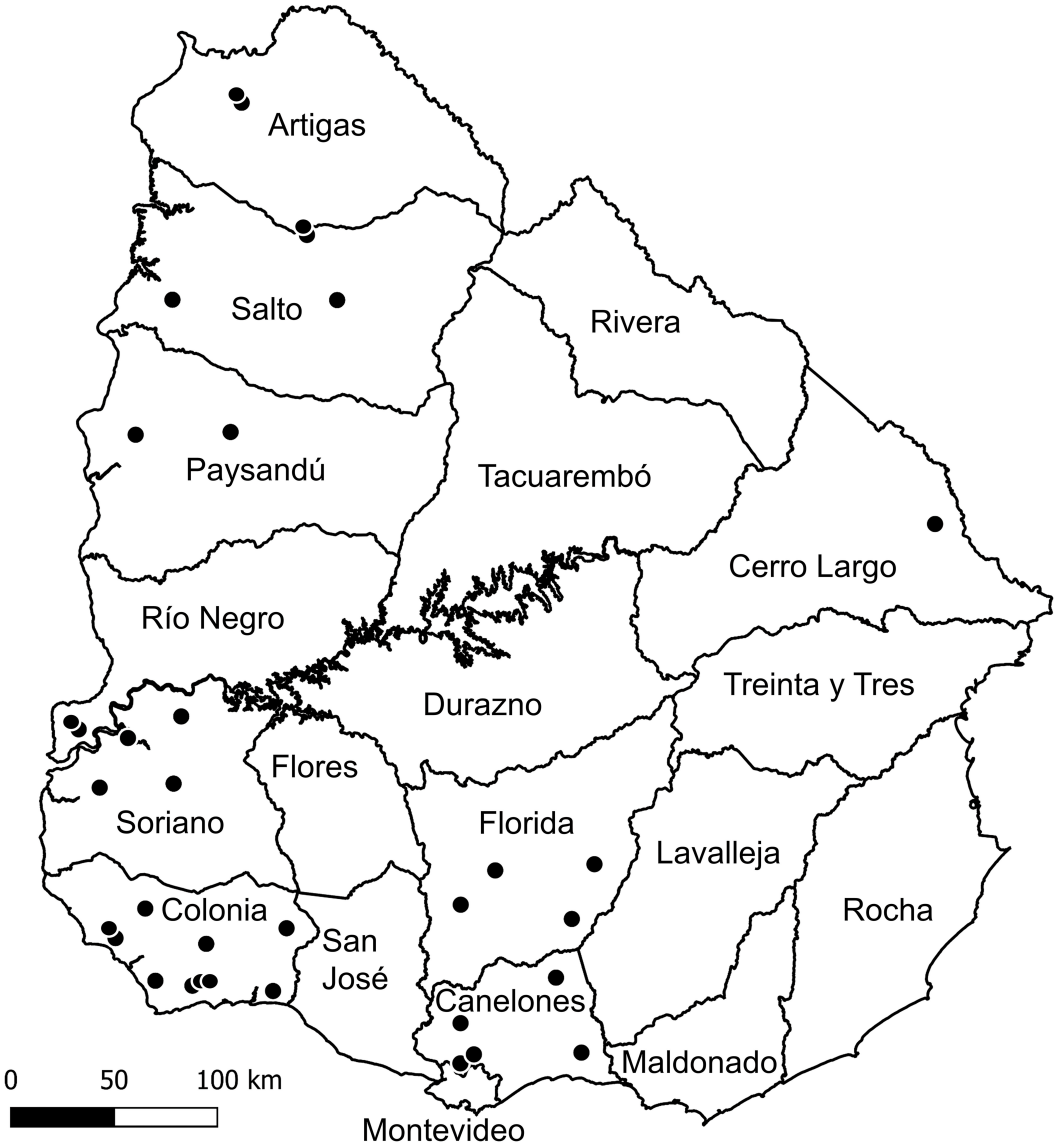
Map of Uruguay showing the geographical location of the 34 farms (black dots) submitting cases by department. The map was created with QGIS software version 2.14 (http://qgis.osgeo.org).

Submissions consisted mainly of aborted fetuses without their placentas (*n* = 58), followed by aborted fetuses with their placenta (*n* = 36) and placentas from aborted sheep without the fetuses (*n* = 6), totaling 100 cases. Eighty-one cases submitted to the laboratory belonged to 61 aborted sheep, which were carrying one (*n* = 30), two (*n* = 24), three (*n* = 6) or five fetuses (*n* = 1) (not all fetuses/placentas expulsed by these aborted ewes were submitted to the laboratory, thus not all of them account for cases). The number of aborted sheep and their fetal loads was unknown for the remaining 19 cases, although because 12 of these cases were submitted on different dates or from different farms, they belonged to at least 12 different sheep. Therefore, the 100 cases belonged to at least 73 aborted sheep. The farms from where cases were submitted were distributed in 9 of the 19 departments (47.4%) Uruguay is geographically subdivided in Figure 1. The geographic location of the farms (departments), the number of farms and cases analyzed for each department and farm are shown in Table 2.

**Table 2:** Geographic location of the farms, number of farms, number of analyzed cases, and identified etiologies in 100 cases of ovine abortion investigated during the 2015–2021 period.

**Department**	**No. of farms (farm ID)**	**No. of cases (cases per farm)^a^**	**Infectious etiologies**			**Dystocia^a^**
			***T. gondii* ^a^**	***Campylobacter fetus fetus* ^a^**	***Campylobacter* sp.^a^^,^^b^**	
Artigas	2 (A–B)	2 (A1, B1)	2 (A1, B1)	0	0	0
Canelones	5 (A–E)	6 (A1, B2, C1, D1, E1)	5 (A1, B2, C1, E1)	0	0	0
Cerro Largo	1 (A)	1	1	0	0	0
Colonia	10 (A–J)	33 (A11, B2, C8, D1, E1, F3, G1, H1, I3, J2)	8 (B2, D1, E1, F2, J2)	1 (C1)	1 (H1)	1 (A1)
Florida	4 (A–D)	32 (A25, B3, C1, D3)	4 (C1, D3)	0	0	12 (A9, B3)
Paysandú	2 (A–B)	5 (A4, B1)	0	0	0	0
Río Negro	2 (A–B)	6 (A2, B4)	2 (A2)	4 (B4)	0	0
Salto	4 (A–D)	7 (A2, B1, C3, D1)	4 (A2, C1, D1)	0	0	0
Soriano	4 (A–D)	8 (A3, B1, C1, D3)	1 (A1)	0	0	0
Total	34	100	27 (58.7%)^c^	5 (10.9%)^c^	1 (2.2%)^c^	13 (28.3%)^c^

*ID, identification*.

a *Capital letters within parentheses indicate each individual farm ID, and the accompanying numbers indicate the number of cases from each farm*.

b *Although the species of Campylobacter could not be identified, specific testing for C. fetus and C. jejuni was negative*.

c *Indicates the percentage of 46 cases with an etiologic diagnosis*.

The gestational age was estimated in the 94 autopsied fetuses: 70 were in the last third of gestation (>100 days), while the remaining 24 where in the second third (50– ≤ 100 days). The degree of autolysis was minimal in 1 case, mild in 35 cases, moderate in 35 cases, and severe in 18 cases; this information was not recorded in the remaining 11 cases. Twenty-three of the 94 (24.5%) fetuses exhibited mummification, all of which had severe (14 fetuses) or moderate (9 fetuses) autolysis. The number of cases submitted from each farm ranged from 1 to 25, and no more than one etiologic diagnosis was attained in a single farm. However, there was evidence of infection/exposure to up to three pathogens in two different flocks (*Leptospira* spp., *T. gondii*, and BVDV-1i in one flock, and *Leptospira* spp., *T. gondii*, and *Cff* in another) by molecular, bacteriological and/or serological methods, although the pathologic examination of cases from these flocks did not reveal lesions attributable to the detected agents, except for *Cff*.

### Combined Interpretation of Results: Pathological Findings and Etiologic Diagnosis

**Figure 2:**
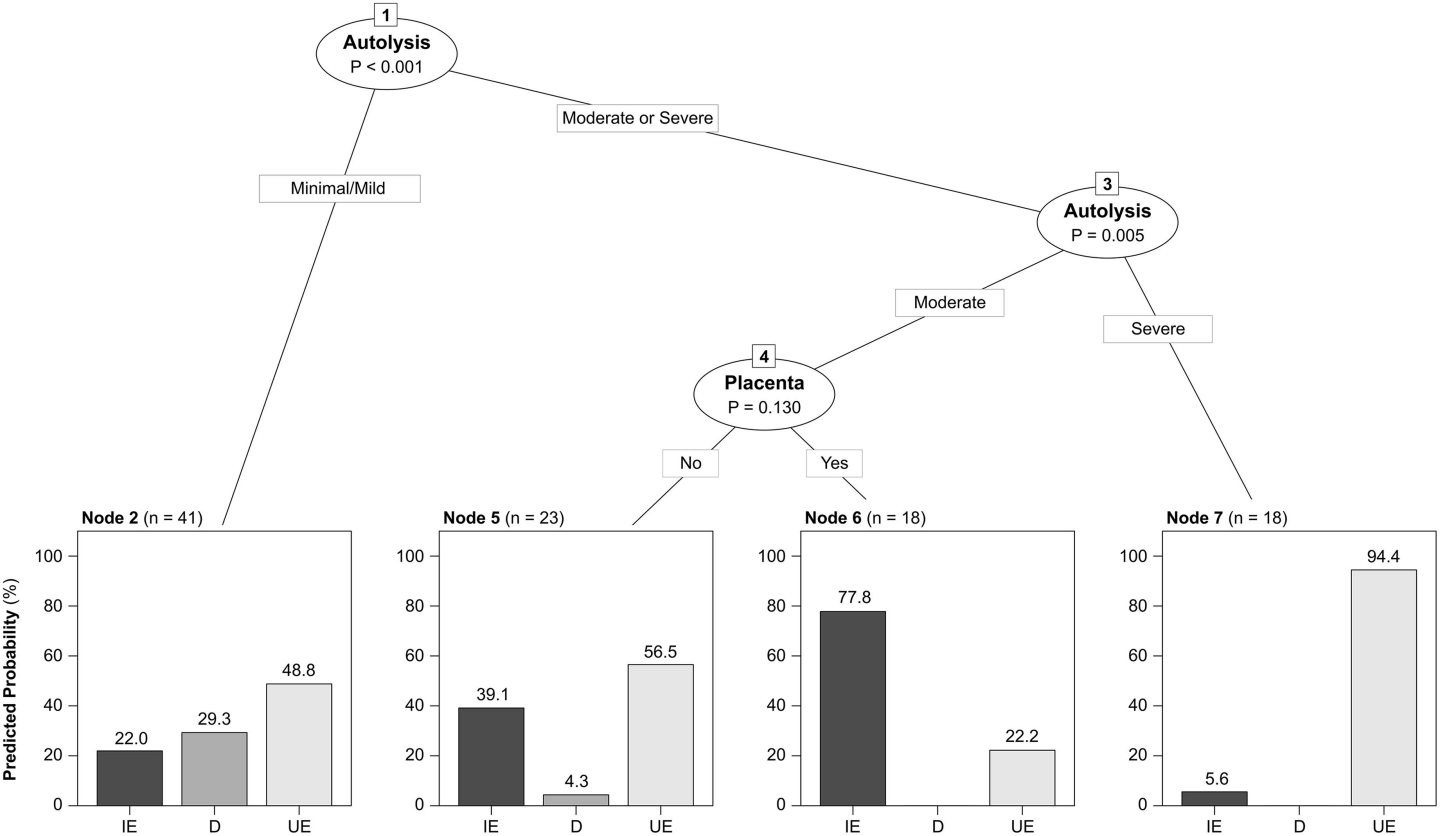
Conditional inference tree fitted by repeated cross-validation showing the predicted conditional distribution of diagnostic categories as a function of the degree of autolysis and the availability of placenta. Node 2 grouped cases with mild/minimal autolysis, in which the predicted probability of reaching an etiologic diagnosis of dystocia was highest. Nodes 5 and 6 grouped cases with moderate autolysis, in which the probability of reaching an etiologic diagnosis of infectious etiology was higher when placenta was available (node 6) versus unavailable (node 5). Node 7 grouped cases with severe autolysis which had higher probability of having an undetermined etiology. IE, Infectious etiologies (*n* = 33). D, Dystocia (*n* = 13). UE, Undetermined etiology (*n* = 54). Fetal mummification was also considered as a potential predictor, but it was not retained in the final tree as it did not improve the classification accuracy.

The etiologic diagnoses are summarized in Table 2. An etiologic diagnosis was attained in 46/100 (46%) cases submitted from 25/34 (73.5%) farms. This frequency varied with the type of submission, being 41.4% (24/58) for fetuses without placentas, 66.7% (4/6) for placentas without fetuses, and 50.0% (18/36) for fetuses with their placentas. Despite this, these differences were not statistically significant (*p* = 0.4). The proportion of cases with an etiologic diagnosis was not significantly different in fetuses with (7/23, 30.4%) or without (35/71, 49.3%) mummification (*p* = 0.1). Additionally, an etiologic diagnosis was reached in 20/36 (55.6%) cases with minimal/mild autolysis, 20/35 (57.1%) cases with moderate autolysis and 1/18 (5.6%) cases with severe autolysis, this percentage being significantly lower in the latter (*p* = 0.0003). The CTREE that best classified the data included the degree of autolysis and the availability of placenta as predictors (Figure 2).

#### Infectious Etiologies

An etiologic diagnosis of abortion with infectious etiology was reached in 33/100 (33%) cases, representing 71.7% of the 46 cases with an etiologic diagnosis (Table 2).

##### Toxoplasmosis

An etiologic diagnosis of toxoplasmosis was reached in 27% of the 100 cases (81.8% of the 33 cases with an infectious etiology), in 19/34 (55.9%) farms from 8 of the 9 departments (Table 2). Of these 27 cases, 14 were fetuses with placenta, 10 were fetuses without placenta, and 3 were placentas without fetuses. Toxoplasmosis was diagnosed in 17/42 (40.5%) cases with placenta and 10/58 (17.2%) cases without placenta, this difference being statistically significant (*p* = 0.01). Seven of the 24 fetuses (29.2%) were mummified. The percentage of cases with an etiologic diagnosis of toxoplasmosis did not differ significantly in mummified (7/23, 30.4%) compared to non-mummified (17/71, 23.9%) fetuses (*p* = 0.5). In mummified fetuses (7/23 cases) and cases with severe autolysis (1/18 cases), toxoplasmosis was the only achieved etiologic diagnosis. Abortions were in the last (13 cases) and second (10 cases) third of gestation, with an average fetal age of ~105 days. No cases were identified in the first third of gestation; the gestational age was unavailable in four fetuses. Delivery of a viable healthy lamb along with the expulsion of a single mummified aborted fetus was recorded in 2 cases.

**Figure 3:**
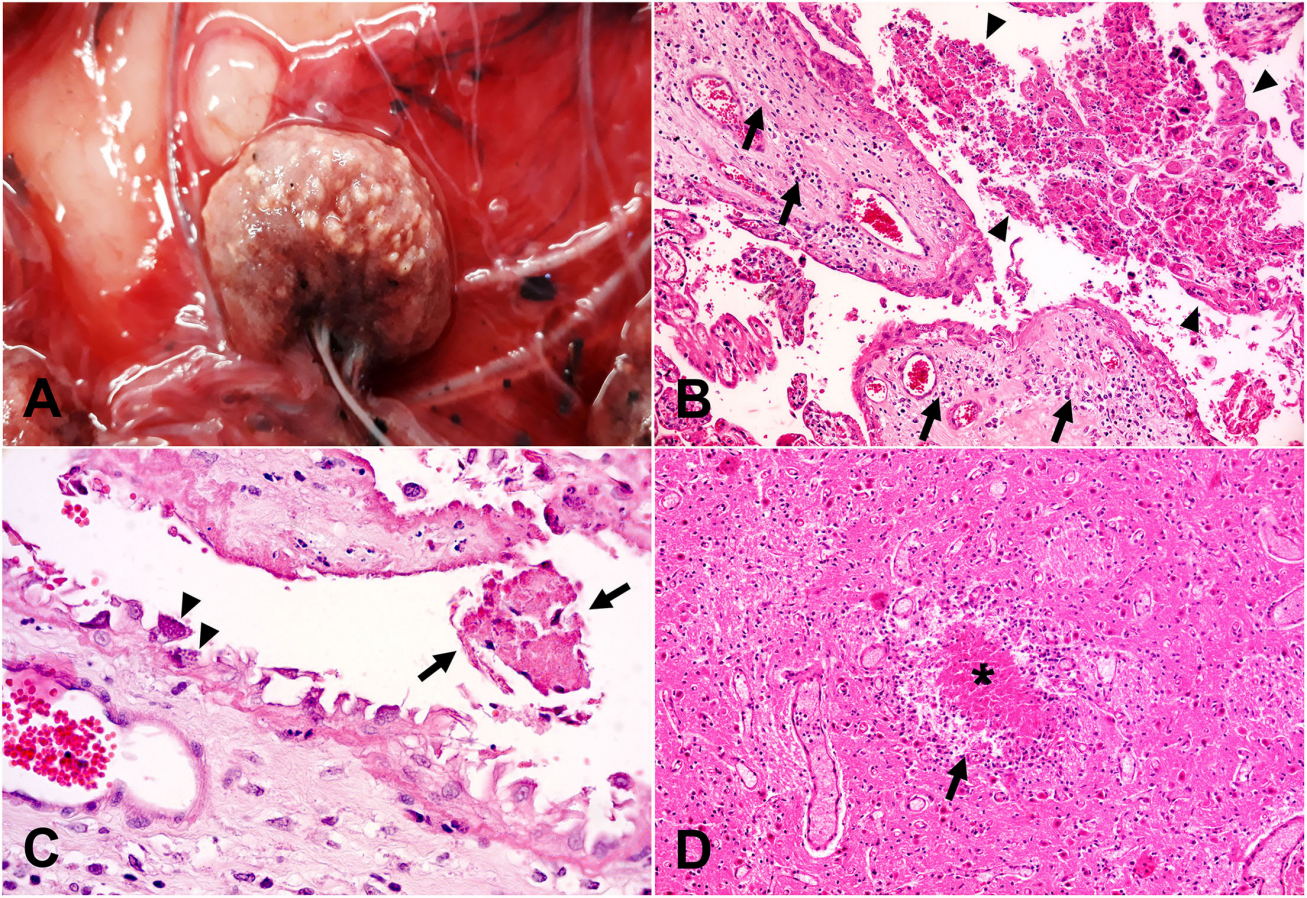
Pathological findings in cases of abortion with etiologic diagnosis of toxoplasmosis. **(A)** Gross view of a placenta (chorionic membrane) depicting a cotyledon (center) containing multiple, well-demarcated round to oval pale whitish foci of ~1 × 2 mm, consistent with foci of necrosis and mineralization (multifocal cotyledonary necrotizing placentitis). **(B)** Histologic correlate of A, extensive trophoblastic necrosis, and sloughing (arrowheads) along with inflammatory infiltrates (arrows) in the chorionic stroma (necrotizing placentitis). Hematoxylin and eosin stain, original magnification 200×. **(C)** Scattered trophoblasts attached to the chorionic stroma contain large numbers of intracytoplasmic pyriform basophilic structures consistent with *T. gondii* zoites (arrowheads) adjacent to an area with sloughed trophoblastic necrotic debris (arrows). Hematoxylin and eosin stain, original magnification 630×. **(D)** Brain, necrotizing encephalitis characterized by a focal area of necrosis (asterisk) surrounded by a rim of glial and inflammatory cells (arrow). Hematoxylin and eosin stain, original magnification 200×.

Generally, the proportion of aborted sheep in flocks with cases of toxoplasmosis was 1–4%, except for two flocks with higher abortion rates. In one of them, 8 of 70 ewes (11.4%) aborted within 15 days, while in the other 4 of 25 ewes (16.0%) aborted within 7-10 days. Typical placental gross lesions, characterized by tan cotyledons with multiple white foci of ~1 × 2 mm were observed in one case (Figure 3A). Regardless of the observation of gross lesions, the histologic examination revealed multifocal non-suppurative and necrotizing cotyledonary placentitis with occasional mineralization in 16/17 (94.1%) cases with placenta available for examination (Figures 3B,C). Fetal tissues exhibited typical lesions of protozoal etiology in 18/24 cases (75.0%). These included multifocal necrotizing encephalitis and gliosis (16 cases, Figure 3D), non-suppurative myocarditis with or without epicarditis (13 cases), glossitis (11 cases), hepatitis (11 cases), skeletal myositis (8 cases), extensive periventricular leukomalacia (6 cases), non-suppurative multifocal interstitial pneumonia (6 cases), adrenalitis (3 cases), and interstitial nephritis (2 cases). Pyriform basophilic structures compatible with protozoal tachyzoites were observed forming clusters inside trophoblastic cells (Figure 3C) in two placentas, and within the cytoplasm of hepatocytes adjacent to necrotic areas in the liver of another case.

In one case with an etiologic diagnosis of toxoplasmosis, BVDV-2b was identified by PCR followed by sequencing. The case consisted of a fetus and its placenta, expulsed at an estimated gestational age of 75 days. It had typical lesions of protozoal etiology including severe multifocal necrotizing non-suppurative encephalitis with gliosis, multifocal non-suppurative myocarditis/epicarditis and myositis, multifocal non-suppurative and necrotizing interstitial pneumonia, multifocal random non-suppurative and necrotizing hepatitis, multifocal non-suppurative necrotizing cortical and medullary adrenalitis, and multifocal cotyledonary and intercotyledonary non-suppurative placentitis with multifocal trophoblastic necrosis and chorionic mineralization. *Toxoplasma gondii* DNA was detected in the brain and placenta. Because of the molecular identification of BVDV-2b, sections of liver, lung, heart, spleen, and kidney were processed by IHC for the detection of BVDV antigen, with negative results in all tissues. A sample of serum of the aborted sheep obtained at the time of the abortion was tested by PCR for the detection of Pestivirus, with a negative result. Altogether, these results indicated a transient fetal infection with BVDV-2b in a nonviremic ewe. The animal was from a small flock of 20 sheep. Notably, there were cattle on the premises.

##### Campylobacteriosis

Abortions caused by *Cff* accounted for 5% of the total cases and 15.2% of the 33 cases with an infectious etiology. This etiologic diagnosis was reached in 2/34 (5.9%) farms from different departments (Table 2). These losses occurred in the last third of gestation, with an average gestational age of 120 days. Four of the cases were fetuses without placenta from one farm, the remainder case represented a placenta from an aborted ewe from another farm. The four fetuses submitted from the same farm were aborted by at least 2 and a maximum of 3 sheep (2 fetuses were twins, the other 2 could have been twins or expulsed by different sheep). In this farm, 20 of ~180 pregnant sheep (11.1%) aborted during the lambing season, while in the other farm, 3 of 57 pregnant ewes (5.3%) aborted within 1 week. *Cff* isolation was achieved in four cases (3 fetuses and the placenta), and detection of *C. fetus* was successful in all 5 cases by qPCR. Additionally, *C. fetus* was identified by DFAT in imprints of lung, liver and/or abomasal fluid smears in 3/4 fetuses (the case consisting of placenta only was not processed by DFAT). The pathologic examination of the fetuses revealed severe multifocal necrotizing and suppurative hepatitis (4 cases), mild lymphocytic myositis (2 cases), severe extensive suppurative bronchopneumonia, mild lymphocytic myocarditis, neutrophilic and histiocytic meningitis, neutrophilic and fibrinous splenitis, neutrophilic enterocolitis and abomasitis with fibrinosuppurative peritonitis and microthrombosis of mesenteric vessels, and neutrophilic and histiocytic mesenteric lymphadenitis (1 case each). In the only case with placenta available for examination, there was severe fibrinonecrotizing placentitis with necrotizing arteriolitis and microthrombosis of chorionic arterioles.

**Figure 4:**
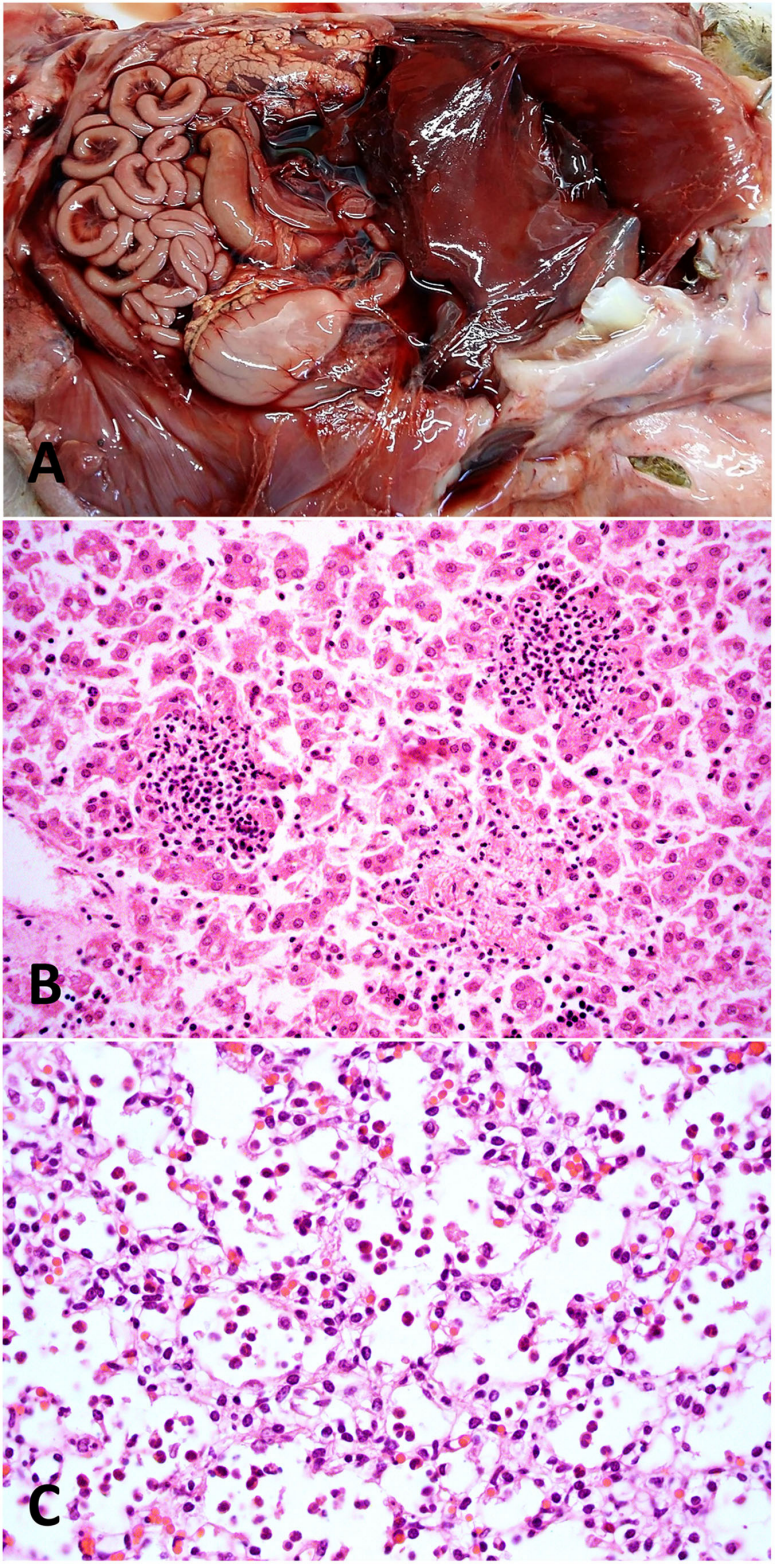
Pathological findings in a fetus with an etiologic diagnosis of campylobacteriosis. **(A)** Abdominal cavity, fibrinous peritonitis and hepatic capsulitis with fibrinous adhesions between the hepatic capsule and the diaphragm. **(B)** Liver, multifocal random necrotizing and neutrophilic hepatitis. Hematoxylin and eosin stain, original magnification 400×. **(C)** Lung, alveolar spaces contain moderate numbers of neutrophils (neutrophilic alveolitis/pneumonia). Hematoxylin and eosin stain, original magnification 400×.

An unidentified species of *Campylobacter* was detected by conventional PCR targeting the 16s rRNA gene in lung and abomasal fluid of a late-term fetus (1% of the cases, and 3% of the 33 cases with an infectious etiology), as we previously reported (24). A 734 bp fragment of the 16s rRNA gene amplified from the abomasal fluid was sequenced and used to assess its similarity with publicly available sequences by BLAST, revealing a species of *Campylobacter* that was 99–100% identical to *C. jejuni, C. coli, C. insulaenigrae*, and *C. hepaticus*, while ruling out *C. fetus*, as we previously described (24). DNA extracted from lung, liver, and abomasal fluid of this fetus tested negative by qPCR for *C. jejuni* and *C. fetus*. DFAT revealed very weak fluorescence of rare, curved bacilli morphologically resembling *Campylobacter* spp. in a liver imprint, but not in imprints of lung and abomasal fluid. No pathogens were isolated on bacterial cultures, including selective cultures for *Campylobacter* spp. Pathological findings consisted of fibrinous polyserositis (Figure 4A), multifocal random necrotizing and neutrophilic hepatitis (Figure 4B), pneumonia with neutrophilic alveolitis (Figure 4C), and erosive necrotizing and neutrophilic enteritis (24). As all these lesions are compatible with campylobacteriosis, an etiologic diagnosis of *Campylobacter* sp. abortion was made, although the causative *Campylobacter* could not be identified at the species level. This abortion was the only one registered during the lambing season in the flock that consisted of 23 sheep (sporadic abortion) in a small commercial/backyard operation.

#### Non-infectious Etiologies (Dystocia)

**Figure 5:**
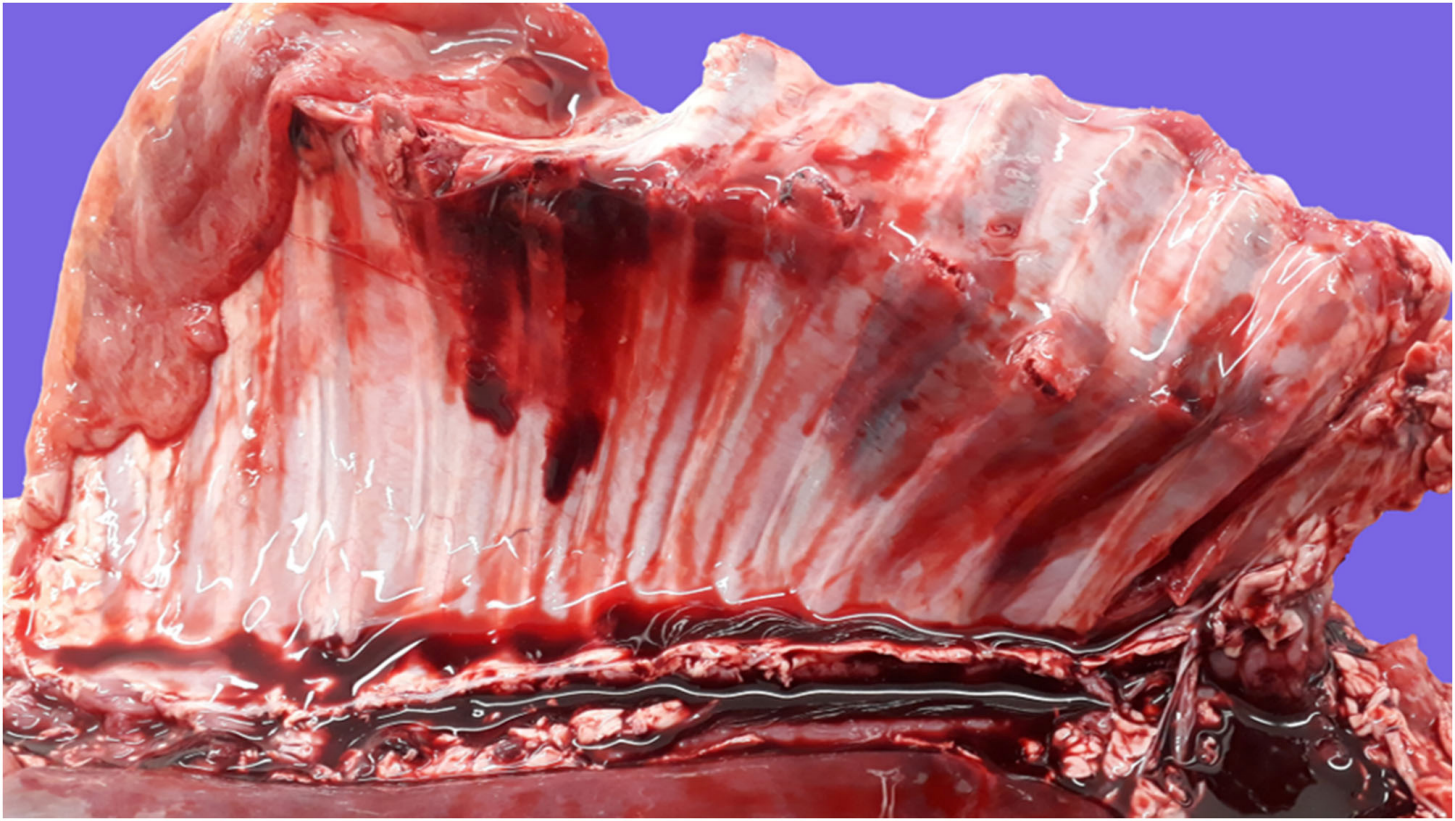
Pathological findings in a full-term fetus with an etiologic diagnosis of dystocia. Serosal view of the right rib cage. Multiple complete displaced fractures of the body of 9 contiguous ribs with associated extensive pleural and subpleural hemorrhages and tearing of adjacent intercostal muscles. There is noncoagulated blood collected in the thoracic cavity (hemothorax), and the diaphragm and intercostal muscles of nonaffected areas are pale, consistent with anemia secondary to exsanguination. These lesions are attributable to severe acute trauma due to dystocia.

Dystocia (Figure 5) was diagnosed in 13 of the 100 cases (13%) from 3 of 34 (8.8%) farms, and 28.3% of the 46 cases with an etiologic diagnosis. All 13 cases of dystocia corresponded to 8 different sheep, 7 of which (87.5%) carried multiple fetuses varying from twins to quintuplets; in the remaining case this information was not available. Six of these 8 sheep (75.0%) were from the same experimental farm and particularly from a highly prolific flock. Dystocia was diagnosed in 9 of 25 cases (36.0%) submitted from this single farm and was the only etiologic diagnosis reached at the farm level, despite being the single farm with the largest number of submissions. The other cases of dystocia were from commercial flocks.

#### Cases of Undetermined Etiology

In 54 of the 100 cases (54%) the criteria to establish an etiologic diagnosis were not met, thus they were considered abortions of undetermined etiology. Fourteen of these cases (14% of the 100 cases, and 25.9% of the 54 cases with undetermined etiology) had inflammatory and/or necrotizing microscopic lesions in at least one tissue (placenta, heart, brain, lungs, kidneys, liver, spleen, adrenal glands, and/or tongue) that were attributable to a pathogenic microorganism. However, either no pathogens were identified by the performed laboratory tests, or the identified infectious agents were opportunists (see below) or could not be causally associated with the observed lesions, therefore all these 14 cases were considered of undetermined etiology.

*Campylobacter jejuni* was detected by qPCR in the liver of an 80-day-old severely autolyzed and mummified fetus. The advanced state of postmortem decomposition precluded the identification of eventual histological lesions, subsequently the criterion to establish an etiologic diagnosis was not met. No bacterial pathogens were isolated from this case, including *Campylobacter* spp., and *C. fetus* DFAT was negative. No other abortions were detected concurrently in this commercial flock which had approximately 100 ewes.

*Leptospira* spp. DNA was detected by the SYBR Green based qPCR in one case. The case consisted of one fetus and its placenta aborted by a sheep carrying triplets at the end of gestation. Although all three fetuses were expulsed dead, unfortunately only one of them and its placenta were submitted to the laboratory for analysis. Despite testing the placenta, kidney, liver, and abomasal fluid, only the placenta yielded a positive result. The amount of *Leptospira* spp. DNA detected was below the limit of quantification of this test, which is 10 genomic copies/μL. DNA extracted from duplicates of the same samples (placenta, kidney, liver, and abomasal fluid) as well as lung, spleen, and brain were processed by the TaqMan^TM^ based qPCR protocol for *Leptospira* spp., with negative results in all samples. All other testing for *Leptospira* spp. (IHC on placenta, lung, and liver; culture on EMJH from kidney, liver, and placenta; and DFAT on abomasal fluid, kidney, and liver) yielded negative results. Additionally, no antibodies were detected by MAT in the fetal fluid (cut-off 1:10) nor in a sample of serum of the aborted sheep (cut-off 1:100) collected at the time of abortion. In this context, and taking all results into consideration, the positive result obtained by the SYBR Green based qPCR for *Leptospira* spp. was disregarded. Histopathologic examination revealed multifocal moderate acute microthrombosis in the chorionic capillaries in the placenta, multifocal mild acute microthrombosis in pulmonary capillaries, and mild canalicular cholestasis in the liver. Incidentally, there was multifocal chronic osseous metaplasia in the chorionic placenta. Although *T. gondii* DNA was detected in the fetal brain, there were no lesions attributed to toxoplasmosis in the brain or any other examined tissue in this case; *T. gondii* PCR was negative in the placenta. As this case did not fulfill the criterion established to achieve an etiologic diagnosis, it was considered an abortion of undetermined cause.

Opportunistic bacteria that have been sporadically associated with ovine abortion in the literature were identified in 2 cases (2%) with lesions suggestive of an infectious etiology. One was a moderately autolyzed, 120-day-old fetus with multifocal, moderate, acute fibrinous pneumonia (alveolitis and bronchiolitis), and multifocal mild canalicular cholestasis in the liver; *Bacillus licheniformis* was isolated on bacterial cultures. The other case was a mummified and moderately autolyzed fetus of 110 days of gestation with multifocal mild neutrophilic meningitis and circulating leukocytosis/neutrophilia in meningeal and cerebrocortical capillaries. *Bacillus licheniformis* and *Streptococcus* sp. were isolated on bacterial cultures, while all other tests to assess for bacterial pathogens were negative. *Toxoplasma gondii* DNA was detected in the brain, but no lesions typical of toxoplasmosis were identified in any of the examined fetal tissues. In both flocks the abortions were sporadic. While these opportunistic bacteria could have been the etiologies of the described lesions in these two fetuses, such lesions could have also been caused by other pathogens that would have been difficult to identify considering the state of postmortem decomposition in both cases. Because these agents are also occasional contaminants, these two cases were classified as abortions of probable infectious cause, of uncertain etiology despite isolating these opportunists.

BVDV-1i was identified by PCR followed by sequencing in one fetus. The sheep carried twins until the end of gestation; one of the lambs was born alive and survived, while the other was expulsed dead and submitted to the laboratory. Abortions were sporadic in this flock. The fetal autopsy revealed mild autolysis, lack of mummification and partial aeration of the lungs, suggesting that the fetus was alive until shortly before expulsion. The histopathologic examination revealed multifocal moderate lymphohistiocytic interstitial nephritis. Because of the molecular identification of BVDV-1i, several fetal tissues including kidney, heart, spleen, liver, and adrenal gland were processed by IHC for the detection of BVDV antigen, with negative results. Additionally, the MAT conducted in a sample of fetal thoracic fluid revealed an antibody titer of 1:80 to *Leptospira* serogroup Pyrogenes, with no titers to all other tested serovars. The DFAT, qPCR and culture for *Leptospira* spp. were all negative in this fetus. A sample of kidney was subjected to IHC for the detection of *Leptospira* spp. antigen, with a negative result.

#### Congenital Malformations

Congenital malformations were identified in three fetuses. One was a partially mummified female fetus of approximately 110 days of gestation with inferior prognathism; this case was the one with neutrophilic meningitis of undetermined etiology from which the opportunistic bacteria *Bacillus licheniformis* and *Streptococcus* sp. were isolated (as described above). Another case with a congenital malformation was a full-term male fetus with a focal, well demarcated, flaccid-walled and fluid-filled cyst of ~2 cm diameter in the diaphragmatic aspect of the left liver lobe (hepatic cyst). Dystocia unrelated to this incidental congenital malformation was the identified cause of fetal death in this case. Another case with a congenital malformation was a 135-day-old, male fetus with complete agenesis of the liver, descendent segment of the duodenum, jejunum, ileum, colon, and cecum. Although this malformation would have been incompatible with postnatal life if the fetus would have survived until the end of gestation, the attributed cause for the abortion in this case was toxoplasmosis, as it had severe extensive non-suppurative necrotizing placentitis with occasional neutrophils and *T. gondii* detected by PCR in the placenta and brain.

### Laboratory Test Results

#### Bacteriological Cultures

Samples from 89 cases were cultured. *Cff* was isolated in 4 cases of campylobacteriosis (see above) being the only primary abortigenic bacterial pathogen isolated. Opportunistic bacteria including *Bacillus licheniformis* and *Streptococcus* sp. were isolated in 2 cases with lesions of undetermined cause (described above). In 43 cases, bacteria that were isolated either in pure or mixed cultures were considered contaminants, including an isolate of *Bacillus cereus* obtained from a fetus without lesions. Other contaminants were *Proteus* spp., *Providencia* spp., *Morganella* spp., *Brevibacterium* sp., *Pseudomonas* spp., *Enterococcus faecalis, Citrobacter* spp., and *Escherichia coli*. Bacteriological culture was negative in 42 cases. None of the cultures yielded *Brucella* spp., *Listeria* spp., *Salmonella* spp., *C. jejuni, Yersinia* spp., or other known bacterial abortifacients of sheep. None of the 86 cases cultured for *Leptospira* spp. in EMJH medium yielded an isolate identifiable by dark field microscopy.

#### Direct Fluorescent Antibody Tests

Fetal tissue and placental imprints and smears of abomasal fluid were examined by DFAT for *C. fetus* and *Leptospira* spp. in 83 and 81 cases, respectively. Strongly fluorescent curved bacilli compatible with *C. fetus* were observed in 3 of 4 fetuses with an etiologic diagnosis of campylobacteriosis by *Cff* (see above). No fluorescent spirochetes morphologically compatible with *Leptospira* spp. were detected in any of the evaluated samples.

#### Molecular Testing

Samples of 91 cases were analyzed by PCRs for Apicomplexa Coccidia, *T. gondii* and *N. caninum*. *Toxoplasma gondii* DNA was detected in 42 cases, including all 27 cases with an etiologic diagnosis of toxoplasmosis described above. In the remaining 15 cases, *T. gondii* DNA was detected but typical lesions of toxoplasmosis were not observed. All cases that tested positive for *T. gondii* were also positive for Apicomplexa Coccidia, and all *T. gondii*-negative cases were also negative for Apicomplexa Coccidia. All cases tested negative for *N. caninum*.

*Campylobacter fetus* and *C. jejuni* qPCRs were performed on 92 cases; 5 tested positive for *C. fetus* (corresponding to the 5 cases of campylobacteriosis described above -4 fetuses and 1 placenta-) and 1 was positive for *C. jejuni*. The latter was a severely autolyzed and mummified fetus as described above. In another case (fetus), an unidentified species of *Campylobacter* was detected as described above and we previously reported (24); the qPCRs for *C. jejuni* and *C. fetus* were negative in this case.

Except for the single case described above, *Leptospira* spp. DNA was not detected in any of the other 88 cases assessed by the SYBR Green based qPCR. Finally, no *C. abortus, C. pecorum* or *C. burnetii* DNA was detected in any of the 85 analyzed cases.

Pestivirus was detected by PCR in 2 of 91 cases. Sequencing revealed BVDV-1i and BVDV-2b in the other (as described above). Border disease virus was not identified in any case.

#### Serologic Testing

The MAT was performed in 63 fetal fluid samples (in many mummified fetuses severe dehydration precluded obtaining this sample). Results are shown in Table 3. Anti-*Leptospira* spp. antibodies were detected in 15 fetuses (23.8%) from 9 of 34 (26.5%) farms.

**Table 3:** Fetal fluid samples seroreactive to microscopic agglutination test (MAT) performed using 12 *Leptospira* spp. serogroups and a cut-off titer of 1:10.

**Case ID**	**Reference serogroups**							**Autochthonous serogroups**				
	**Can**	**Bal**	**Gri**	**Ict**	**Pom**	**Tar**	**Sej**	**Sej**	**Pyr**	**Aus**	**Aut**	**Pom**
19-088 A	–	–	–	1:20	–	–	–	–	–	–	–	1:160
19-088 B	1:10	1:10	1:10	1:40	1:160	1:80	1:20	1:80	1:40	1:20	1:20	1:160
19-093 A	1:80	1:20	–	1:20	–	–	–	–	1:10	–	–	–
19-099	1:40	–	1:20	1:20	1:10	–	–	–	–	–	–	–
19-100 A	–	–	–	–	1:80	–	1:20	–	1:40	1:80	–	1:10
19-105	–	–	–	–	–	–	–	–	1:80	–	–	–
19-116	1:20	–	–	–	–	–	–	–	–	–	-	-
19-119 A	1:20	–	–	–	–	–	–	–	–	–	–	–
20-047 C	–	1:10	–	–	–	–	–	–	–	–	–	–
20-047 D	–	–	–	1:10	–	–	–	–	–	–	–	–
20-061 A	–	–	–	1:10	–	–	–	–	–	–	–	–
20-061 B	1:10	1:20	-	1:10	–	–	–	–	–	–	–	1:10
20-061 C	1:10	1:10	-	1:10	–	–	–	–	–	–	–	–
20-064 B	–	–	–	–	1:10	–	–	1:10	–	–	1:10	1:80
20-066	–	–	–	–	–	–	–	1:40	–	–	1:10	–
Total	7	5	2	8	4	1	2	3	4	2	3	5

## Discussion

Herein we identified causes of fetal losses and their relative frequency in sheep flocks in Uruguay. To our knowledge, this study represents the largest case series conducted in South America, contributing to deepening the regional understanding of ovine abortion-causing agents.

The overall diagnostic efficiency (percentage of cases with an etiologic diagnosis) in this series was similar to those reported in previous studies (8, 18). Diagnostic efficiency was not significantly influenced by the type of submission, though it was slightly higher when placental samples were included, in agreement with other studies (10, 12–14). This reinforces the value of the placenta in the diagnostic investigation of abortion, particularly for infectious diseases, as highlighted before (14, 46–48).

In our study, advanced autolysis reduced the probability of reaching an etiologic diagnosis (49, 50). This is partially related to the criteria established to attribute abortion causality in cases with an infectious etiology, which included the co-identification of lesions and pathogens. Because advanced autolysis affects both the ability to recognize eventual tissue lesions, as well as detecting pathogens, it comes to no surprise that the percentage of etiologic diagnoses was significantly lower in cases with severe autolysis. However, in many cases with moderate autolysis and even in mummified fetuses, the diagnostic criteria we established were fulfilled, most notably in cases of toxoplasmosis. As a cyst-forming protozoon, *T. gondii* is resistant and detectable by PCR in fetal tissues and placentas even in the face of moderate autolysis / postmortem decomposition. Likewise, tissue lesions induced by this parasite can be so severe that they can still be recognized in cases with relatively poor tissue preservation, particularly the encephalitis and to a lesser extent placentitis typical of toxoplasmosis can be reliably detected by experienced pathologists even in the face of some degree of autolysis. This highlights that analyzing all specimens is worthwhile regardless of autolytic changes or mummification status, especially when toxoplasmosis is suspected. The ability to identify the cause of abortion in cases with bacterial or viral etiologies seems to be negatively impacted by autolysis.

Overall, the etiologies identified in this work were those that usually induce recognizable lesions and whose identification is relatively straightforward (8). Consistently, the main infectious agents herein reported (*T. gondii* and *Campylobacter* spp.) are considered major causes of abortion in several countries, such as Australia (12, 18), Canada (51), the USA (8), the Netherlands (10, 14), and New Zealand (19). Additionally, dystocia was identified as a cause of fetal death in 28.3% of the cases with an etiologic diagnosis, similar to a previous work (18), but certainly higher than most case series, in which fetal deaths due to dystocia were in the range of 2–4% (8, 12, 52).

The high frequency of dystocia herein described can be probably explained because 9 of the 13 cases were submitted from one same farm; therefore, this value is biased. This was an experimental farm with a highly prolific flock (high rate of pregnancies with fetal loads ≥2), with improved nutritional management of sheep carrying multiple fetuses in the last third of gestation, determining a high lamb birthweight, which could explain the high rate of dystocia (53, 54). Most of the diagnoses of dystocia in our series involved sheep carrying multiple lambs. It is worth noting that even though this was the farm that contributed with most cases in the series (25/100 cases), dystocia was the only etiologic diagnosis at the farm level. This highlights the higher relative impact of dystocia over infectious diseases as a cause of fetal losses in this flock. Although selection for prolificity increases productivity (i.e., number of weaned lambs per ewe), the higher incidence of dystocia can be of concern for animal welfare.

An etiologic diagnosis could not be achieved in a large number of cases (54%), this is in agreement with the success rates reported in previous case series studies (8, 14, 16, 50). The proportion of cases of abortion of undetermined etiology exhibiting lesions compatible with an infectious agent (14%) was similar to that described by Kirkbride (8). Several causes could account for the failure in identifying abortigenic pathogens, particularly bacteria, including: (1) bacteria that do not grow under standard conditions or have fastidious growth requirements, (2) decreased bacterial viability due to fetal autolysis or mummification, or (3) culture contamination by saprophytic microorganisms that could mask pathogen growth (10). Additionally, there are known infectious abortifacients whose presence in Uruguay is unknown, such as *Francisella tularensis* (55) or Schmallenberg virus (56, 57), for which we did not perform specific testing. Furthermore, the occurrence of unknown abortigenic agents in the region should not be disregarded, as little is known about the spectrum of infectious diseases affecting livestock in the area. On the other hand, abortions without histologic lesions and/or those with congenital malformations could be associated with non-infectious agents such as toxic plants or plants with high phytoestrogen content, deficiencies of trace elements (copper, selenium, iodine, etc.), genetic/hereditary diseases, stress, extreme climatic conditions or adverse reactions to medications and/or vaccines (17, 48). Laboratory identification of these causes is usually difficult or even impossible in most cases (17).

*Toxoplasma gondii* was the most frequent etiology identified in our series, which emphasizes the relevance of this pathogen. It had been previously identified as an ovine abortifacient in Uruguay, first indirectly through a serological survey (58), and then by isolation of the parasite from fetal and placental samples (22, 59). A more recent serological survey performed in flocks in northern Uruguay reported that the seroprevalence of ovine toxoplasmosis is high (26). Accordingly, we diagnosed abortions by *T. gondii* in eight of the nine departments included in the survey. Overall, this evidence that both the parasite and the clinical disease it causes are widely distributed throughout the country.

The severity of clinical toxoplasmosis is strongly dependent on the gestational stage at which infection occurs; during the early stages, infection is usually fatal, but as gestation progresses, premature births, stillbirths and lambs born clinically healthy but congenitally infected are more frequent (60, 61). In addition, it is likely that genetic variability among the different populations of the parasite influences the clinical presentation, since atypical strains (i.e., strains with particular genotypic profiles that differ from the clonal types I, II, and III, and that are widespread in South America) are usually more virulent than clonal type II, which is the predominant lineage in Europe (62, 63). Further studies on the genetic diversity of *T. gondii* infecting sheep in Uruguay are warranted.

In this study, *T. gondii* DNA was detected in 42 cases, 15 of which (35.7%) lacked histological lesions compatible with toxoplasmosis; hence, these were considered subclinical congenital infections. The birth of a healthy lamb accompanied by a mummified fetus, which has been described in cases of ovine toxoplasmosis (9, 60) was registered in two cases in our series. Although there is scarce evidence about the rate of congenital transmission, it is believed that <2% of viable lambs are born infected and <4% are able to transmit the infection to the next generation (64). However, the rate of subclinical congenital infections may be higher than previously thought (65, 66), which would allow the transgenerational transmission and maintenance of the parasite in the flock. It is noteworthy that the main transmission route for sheep is through the ingestion of oocysts excreted by domestic cats and wild felids, of which a few species present in Uruguay have been confirmed as definitive hosts, including pumas (*Puma concolor*), ocelots (*Leopardus pardalis*), and Geoffroy's cats (*Oncifelis onchofelis geoffroyi*) (67). Felids can become infected following predation of infected birds and small mammals (rodents and rabbits), although data on the relevance of intermediate hosts as sources of infection for felids is lacking (68). Further epidemiological studies are needed to better understand the domestic and sylvatic life cycles of *T. gondii* in Uruguay, as well as the role of animals as sources of human toxoplasmosis.

Considering the abortion rates in most farms with an etiologic diagnosis of toxoplasmosis (2–5%), the disease is probably endemic in most farms (47). However, abortion outbreaks/storms (>10%) were registered in yearlings in two farms. As opposed to adult ewes, which develop a long lasting cellular immunity after abortion, yearlings/ewe lambs are the most susceptible category as they usually lack specific anti-*T. gondii* immunity by the time they reach their first pregnancy and therefore, they are more prone to aborting following primoinfection (68). Vaccination is a valuable tool to prevent abortion outbreaks by *T. gondii* in sheep; unfortunately, there are no vaccines currently approved for use in the country.

Macroscopic placental lesions were observed in only one case of toxoplasmosis, perhaps because they are only evident in well-preserved samples (8). Histological findings were consistent with previous descriptions, being the placenta, brain, heart, and skeletal muscle the most frequently affected tissues (68, 69). *Toxoplasma gondii* tachyzoites were rarely seen, and they usually colocalized with lesion sites, as described previously (8). As opposed to what has been suggested by van den Brom et al. (14), the high frequency of lesions we observed in the myocardium demonstrates the value of this tissue for the diagnosis of ovine abortion. In addition, the myocardium is a target organ not only for protozoa, but also for pestiviruses (70, 71).

Given that the microscopic lesions caused by *T. gondii* are similar to those caused by other related protozoa such as *N. caninum* or *Sarcocystis* spp., the diagnosis of abortion by these protozoa requires additional testing (72, 73). In this study, three PCR protocols for the detection of Apicomplexa Coccidia, *T. gondii* and *N. caninum* DNA (37, 38) were implemented. The absence of *N. caninum* DNA in all the tested samples is consistent with a serological survey conducted in 2008 in 10 randomly-selected sheep flocks in three departments of Uruguay (Artigas, Salto, Canelones), which indicates a low *N. caninum* seroprevalence in this species (26). These findings are surprising for several reasons: (1) neosporosis is widely distributed in cattle herds in Uruguay, and is the main infectious cause of abortion identified at the laboratory level (31, 74); (2) *N. caninum* was recently reported to be a cause of abortion in flocks in the central region of Argentina, which shares similar productive, geographical, and climatic conditions with Uruguay (75); and (3) the common utilization of shepherd and/or guardian dogs in sheep flocks in Uruguay, which warrants a close contact between the protozoan's definitive hosts (canids) and the susceptible intermediate hosts (ruminants). The extensive conditions sheep are usually reared under (76), the low number of animals per flock (77) or the pasture-based instead of concentrate-based diets (78), could account for the low prevalence of neosporosis.

Although no specific assay was performed to confirm or rule out the presence of *Sarcocystis* spp., schizonts containing merozoites arranged in a “rosette pattern” are usually histologically evident in fetal tissues, particularly in glomerular capillaries, endothelial cells of the brain and lungs (79–81). No schizonts resembling *Sarcocystis* spp. were observed in our cases. In addition, the PCR for Apicomplexa Coccidia was only positive in cases that were also PCR-positive for *T. gondii*, but in none of the *T. gondii*-negative cases, suggesting that no other Apicomplexa Coccidia were involved. *Sarcocystis* appears to be an extremely infrequent cause of abortion in sheep, since it has been scarcely reported (79–81).

*Campylobacter fetus* subsp. *fetus* was the second most frequent cause of abortion we identified, an agent that had not been detected as an ovine abortifacient in Uruguay until recently (23). Four fetuses were submitted from a flock with a history of sporadic abortions every season. However, the occurrence of multiple abortions in a short period of time had not been observed in this flock until this episode was investigated. This cyclical pattern is characteristic of campylobacteriosis; in endemic farms, abortion storms usually occur every 4–5 years, even though sporadic abortions may be observed every year (47, 82, 83). A possible explanation for this pattern is that flock immunity increases after an abortion outbreak, and wanes over time (47, 82, 83). These observations suggest that asymptomatically infected animals might be a source of infection for susceptible ewes (82).

Grossly, the fetuses with *Cff* infection had rather nonspecific lesions such as increased amount of fluid in the thoracic and peritoneal cavity, or placentitis, as previously described (48, 83, 84). The most striking gross lesion in *Campylobacter* spp. abortions consists of multiple circumscribed, rounded, yellow-grayish foci ranging from a few millimeters to up to 4 cm in diameter, randomly distributed in the fetal liver (8, 48, 85, 86). This lesion was not evident in any of the four fetuses, as reported before (84, 87). Considering that gross hepatic lesions can only be observed in up to 25% of cases (88), and it is not a pathognomonic finding (i.e., *Flexispira rappini* and *Listeria* spp. can cause similar lesions) (8, 89), the etiologic value of this finding seems to be limited, although, when present, it is highly suggestive of a bacterial cause.

A sporadic abortion by an unidentified *Campylobacter* sp. was diagnosed in a flock of 23 ewes. Grossly the fetus had fibrinous peritonitis, a lesion highly suggestive of a bacterial etiology. The agent was detected only by molecular assays; bacterial isolation could not be achieved probably due to its fastidious growth requirements (88), non-optimal incubation temperature (37°C instead of 42°C which is the optimal temperature for thermophilic *Campylobacter* species) (90) or to the advanced degree of fetal autolysis that can compromise bacterial viability. Even though the species could not be confirmed, the most frequently implicated species different from *Cff* is *C. jejuni*, a bacterium commonly associated with abortions in sheep and goats (88). Nevertheless, *C. jejuni* was not detected by a species-specific qPCR in this case. The significance of *C. jejuni* as an ovine abortifacient differs across regions. In New Zealand and Great Britain it is an important cause, and in the USA it has become the main *Campylobacter* species associated with ovine abortion (88, 91). Regarding the other possible species involved in our case, as determined by 16s rRNA gene sequencing (*C. coli, C. insulaenigrae* or *C. hepaticus*), only *C. coli* has been infrequently associated with abortion in sheep (12, 92, 93).

In our series, *C. jejuni* was detected only once in a mummified fetus aborted by a yearling in a flock of 100 sheep where no other abortions were registered. Although autolysis precluded the identification of eventual lesions, its detection in an aborted fetus indicates that *C. jejuni* should be considered a probable cause of abortion in sheep in Uruguay (21). To the best of our knowledge, *C. jejuni* was identified as an ovine abortifacient only recently and once in South America (Argentina) (94).

Both *Leptospira* spp. and BVDV have been associated with reproductive failure in sheep (95–98). In this study, antibodies against different *Leptospira* spp. serogroups were detected by MAT. Amongst them, Pomona, Grippotyphosa and Icterohaemorrhagiae have been implicated in abortions, stillbirths, and death of weak lambs elsewhere (96, 98). Although serological findings should be interpreted with caution given the possibility of cross-reactions, the infecting serogroups are usually identified because of the highest antibody titers (90). Serogroups Pomona, Icterohaemorrhagiae, Canicola, Ballum, and Pyrogenes were the most prevalent here. Among these serogroups, Pomona, Canicola and Pyrogenes had already been reported in cattle in Uruguay (99), as well as in goats and sheep in Brazil (100, 101), evidencing a wider distribution in the region. Although clinical disease by *Leptospira interrogans* serogroup Pomona serovar Kennewicki has been identified in sheep in Uruguay (32), the role of *Leptospira* spp. as a cause of fetal losses remains unknown in this species. Information on *Leptospira* spp. as an ovine abortifacient in South America is currently limited to one report from Argentina. That report described detection of *Leptospira* spp. by molecular (qPCR targeting the 16s rDNA gene) and DFAT in three aborted fetuses with mummification (2 cases) and inflammatory histologic lesions compatible with an infectious etiology (3 cases) (94). The three cases were from two different flocks, but the *Leptospira* species and serogroups involved were not identified. Of note, as opposed to the gene targeted in our study (*lipL32*) which is supposedly present only in all pathogenic *Leptospira* spp. known to date (102), the 16s rDNA gene targeted in the study from Argentina is shared by pathogenic and saprophytic species of *Leptospira*.

In one case in our series, a serological titer of 1:80 against the Pyrogenes serogroup was found in a fetus with lymphohistiocytic interstitial nephritis and autopsy findings indicating that the fetus was alive until shortly before expulsion. Despite this serological titer, *Leptospira* spp. could not be detected by any other ancillary test (DFAT, qPCR, culture on EMJH, and IHC), ruling out an active infection at the time of expulsion; thus, an etiologic diagnosis was not reached. Since it was a late term fetus and therefore capable of eliciting an effective immune response, we hypothesize that the leptospiral infection was successfully controlled in utero, and that fetal death at the end of gestation might have been related to another unidentified cause. Finding MAT titers to one or more *Leptospira* serovars without concurrent identification of leptospires is not uncommon in aborted ovine and bovine fetuses (52). Although likely, whether the nephritis observed in this fetus was a sequela of in utero exposure to *Leptospira* was undetermined. Additionally, BVDV-1i was detected in tissues of this fetus, suggesting an active although possibly subclinical infection, since no lesions typically caused by pestiviruses (encephalitis, myocarditis, cerebellar dysplasia, or other congenital malformations of the brain) were found in the fetus.

In another case, low concentrations of leptospiral DNA (<10 genome equivalents/μl) were detected by the SYBR Green qPCR procedure in the placenta of a full-term fetus. This result, however, was not further confirmed by a second TaqMan^TM^ based qPCR. Given that the rest of ancillary tests, including culture, IHC and MAT in the fetus and dam were all negative, the result yielded by the SYBR Green qPCR was disregarded. The discordant results of both qPCR procedures can be explained by differences in their lower limits of detection (which is higher for the TaqMan^TM^ based procedure), as well as in their sensitivity and specificity. Altogether, even though we performed a large panel of diagnostic tests to assess for this pathogen, its role as an abortifacient could not be confirmed in any case applying the criteria we established for attributing causality. This aligns with the findings of largest ever case series of abortion/stillbirth carried out in sheep, where *Leptospira interrogans* was identified in an extremely low frequency of 1 of 702 (0.14%) cases with an infectious etiology (8).

The two BVDV species and subtypes (1i and 2b) identified here could not be causally associated with the fetal losses according to the diagnostic criterion we set; however, their detection demonstrates active BVDV circulation in pregnant sheep, which is unprecedented in Uruguay. In this context, BVDV should be considered a probable cause of ovine abortion in the country (21). Furthermore, the immunosuppressive properties of BVDV predispose to secondary infections by opportunistic agents (8, 103). Regarding the source of infection, viral transmission among ruminant species can take place when sheep and cattle are kept in the same paddocks or by close contact between the species (104, 105). Both cattle and sheep can be born persistently infected with BVDV and cross species transmission can occur (106). Interestingly, in one farm sheep and cattle coexisted. In the other farm, sheep had grazed on a paddock previously occupied by cattle and they had potential contact with cattle through a fence line separating two properties.

Both BVDV-1i and −2b had been described in cattle in Uruguay, showing a low prevalence in comparison to the dominant BVDV-1a strain (39). Subtype 1i has been detected in cattle in the United Kingdom (107), USA (108), and Brazil (109), but to the best of our knowledge it had never been detected in sheep. Reports of BVDV-2b in cattle suggest that it is more prevalent and more widely distributed in the region than BVDV-1i (39, 110, 111). This finding opposes to what is exposed in a recent meta-analysis where the seroprevalence of BVDV-1 in sheep and goats was higher (112). This difference could be because this meta-analysis only included two studies from South America. Recently, BVDV-2b was identified as a cause of abortion in flocks in Spain as a result of spontaneous infections in one case (97) and the administration of a modified live vaccine against orf virus (Parapoxvirus) containing cattle-derived products contaminated with pestivirus in the other (95).

Despite serological evidence that *C. burnetii* circulates in sheep and cattle in Uruguay (113), and the recent confirmation of cases of abortion in dairy cattle (114, 115), this pathogen was not detected in any of the cases analyzed in our series. Currently, the impact of Q fever in sheep flocks is unknown both at a local and regional level. Similarly, *C. abortus* or *C. pecorum* DNA was not detected in any case. The scarce evidence on the presence of *C. abortus* in Uruguay consists of one serological survey in sheep, in which only 3 individuals (from two different farms) of 107 (2.8%) were positive (27). In contrast, abortions caused by *C. abortus* and *C. pecorum* have been reported in goats in Chile (116) and Argentina (117). The scant available information in Uruguay and neighboring countries suggests that the prevalence of chlamydial enzootic abortion is significantly lower when compared to European flocks, where it is regarded as a major cause of reproductive failure (10, 13, 14, 16, 17, 118).

Several opportunistic bacteria such as *E. coli, Fusobacterium* spp., *Streptococcus* spp., *Staphylococcus* spp., *Bacillus* spp., and *Pseudomonas* spp. can cause sporadic abortion in sheep (8, 16, 48, 50). In this work, opportunistic bacteria were associated with lesions in two cases, but their causative role in the abortion was undetermined (8). Nevertheless, it is likely that opportunistic abortions may be underreported (12, 15, 119).

## Conclusions

This work provides valuable information about the causes of ovine abortion in Uruguay, expanding the body of evidence on a major worldwide health problem of sheep. As reported in other countries, toxoplasmosis and campylobacteriosis were the most frequently identified infectious causes; besides their reproductive impact in sheep, they are zoonotic pathogens of public health concern. The lack of identification of other well-known abortigenic agents, some of which have been reported in local cattle herds, such as *N. caninum* and *C. burnetii*, was somewhat unexpected. In some flocks, dystocia may be a major contributor to fetal losses over infectious diseases. The information contained in this article may help delineate strategies to prevent and control reproductive losses in sheep in Uruguay. Further multidisciplinary studies on the epidemiology of diseases and factors contributing to ovine reproductive losses are needed in the region, including the search of additional causes that may have been unnoticed. The monitoring of fetal losses in sheep enhances surveillance of zoonotic diseases with potential public health impact.

## Data Availability Statement

The raw data supporting the conclusions of this article will be made available by the authors, without undue reservation.

## Ethics Statement

Ethical review and approval was not required for the animal study because it is a study conducted postmortem on natural cases of abortion. Written informed consent for participation was not obtained from the owners because the study used ovine abortion material submitted for diagnostics.

## Author Contributions

MD: collection of data, fetal autopsies, placental examinations, sample collection, photograph acquisition, histopathological and immunohistochemical examinations, integration and interpretation of results, and writing of first manuscript draft. MF, LT, FGo, and AC: molecular testing for *T. gondii* and *N. caninum*. LC, MS, and MB: molecular testing for *Campylobacter* spp. LZ and CC: molecular and serologic testing for *Leptospira* spp. LM, MC, and SM: molecular virology assays. CS: direct fluorescent antibody testing for *C. fetus* and *Leptospira* spp. AR: molecular testing for *Chlamydia* spp. and *C. burnetii*. RC, BD, VA, and CM: fetal autopsies, placental examinations, and sample collection. VA: photograph acquisition. MF: supervised bacteriological cultures. JA: performed statistical analyses. VA, SS, and SF: case acquisition in the field. FGi: histopathological and immunohistochemical examinations, microphotograph acquisition, integration and interpretation of results, coordination of laboratory activities, and manuscript writing and edition. SF and FGi: conceptualized the study. SF, LZ, and FGi: acquired funding. All authors made contributions to the conception and design, read, and approved the final version of the manuscript.

## Funding

This research was financially supported by the Uruguayan Instituto Nacional de Investigación Agropecuaria (INIA, grant PL_27 N-23398) and the Agencia Nacional de Investigación e Innovación (ANII, grants FCE_3_2018_1_148540 and FSA_1_2018_1_152689). MD acknowledges support from INIA through a graduate scholarship.

## Conflict of Interest

The authors declare that the research was conducted in the absence of any commercial or financial relationships that could be construed as a potential conflict of interest. The handling editor GC declared past co-authorships with the authors MD, CS, RC, VA, JA, and FGi.

## Publisher's Note

All claims expressed in this article are solely those of the authors and do not necessarily represent those of their affiliated organizations, or those of the publisher, the editors and the reviewers. Any product that may be evaluated in this article, or claim that may be made by its manufacturer, is not guaranteed or endorsed by the publisher.
