# Supplementary material for: Diagnostic Investigation of 100 Cases of Abortion in Sheep in Uruguay: 2015–2021
Source: Front Vet Sci. 2022 May 19;9:904786. doi: 10.3389/fvets.2022.904786 (PMC9161216; doi:10.3389/fvets.2022.904786)
Supplement: Supplementary file 1 [file Data_Sheet_1.docx]

**Supplementary Materials**

**Immunohistochemistry**

BVDV IHC was performed following a previously described procedure (29), using the mouse monoclonal antibody 15C5 directed against an epitope of a 48-kD glycoprotein of BVDV as the primary antibody. Horseradish peroxidase (HRP) labeled anti-mouse polymer produced in goat (EnVision+ System HRP, K4000, Dako, CA, USA) was used as the detection system and 3-amino-9-ethylcarbazole (AEC) (K3464, Dako, CA, USA) as the chromogen. For the *Leptospira* spp. IHC procedure, the endogenous peroxidase was blocked with 3% hydrogen peroxide for 10 min, a rabbit multivalent antibody (LEP-FAC, NVSL, Ames, Iowa, USA) was applied as a primary antibody as previously described (30) at a dilution of 1:5,000 with no antigen retrieval. HRP labeled anti-rabbit polymer produced in goat (EnVision+ System HRP, K4003, Dako, CA, USA) and AEC (K3464, Dako, CA, USA) were used as the detection system and chromogen/substrate solution. Appropriate positive and negative controls were used for both IHCs. Positive controls consisted of tissues containing the pathogens, while negative controls consisted of serial sections of the same tissues in which the primary antibody was replaced by non-immune serum of the same animal species. All runs were validated by the observation of immunoreactivity in the positive controls and the lack thereof in the negative controls.
